# Recent advances in PGPR-mediated resilience toward interactive effects of drought and salt stress in plants

**DOI:** 10.3389/fmicb.2023.1214845

**Published:** 2023-09-27

**Authors:** Ahmad Al-Turki, M. Murali, Ayman F. Omar, Medhat Rehan, R.Z. Sayyed

**Affiliations:** ^1^Department of Plant Production and Protection, College of Agriculture and Veterinary Medicine, Qassim University, Buraydah, Saudi Arabia; ^2^Department of Studies in Botany, University of Mysore, Mysore, India; ^3^Department of Plant Pathology, Plant Pathology, and Biotechnology Lab. and EPCRS Excellence Center, Faculty of Agriculture, Kafrelsheikh University, Kafr El-Sheikh, Egypt; ^4^Department of Genetics, College of Agriculture, Kafrelsheikh University, Kafr El-Sheikh, Egypt; ^5^Department of Microbiology, PSGVP Mandal’s S I Patil Arts, G B Patel Science and STKV Sangh Commerce College, Shahada, India; ^6^Faculty of Health and Life Sciences, INTI International University, Nilai, Negeri Sembilan, Malaysia

**Keywords:** antioxidants, drought stress, glycine-betaine, osmolyte, oxidative stress, proline, PGPR, salt stress

## Abstract

The present crisis at hand revolves around the need to enhance plant resilience to various environmental stresses, including abiotic and biotic stresses, to ensure sustainable agriculture and mitigate the impact of climate change on crop production. One such promising approach is the utilization of plant growth-promoting rhizobacteria (PGPR) to mediate plant resilience to these stresses. Plants are constantly exposed to various stress factors, such as drought, salinity, pathogens, and nutrient deficiencies, which can significantly reduce crop yield and quality. The PGPR are beneficial microbes that reside in the rhizosphere of plants and have been shown to positively influence plant growth and stress tolerance through various mechanisms, including nutrient solubilization, phytohormone production, and induction of systemic resistance. The review comprehensively examines the various mechanisms through which PGPR promotes plant resilience, including nutrient acquisition, hormonal regulation, and defense induction, focusing on recent research findings. The advancements made in the field of PGPR-mediated resilience through multi-omics approaches (*viz.*, genomics, transcriptomics, proteomics, and metabolomics) to unravel the intricate interactions between PGPR and plants have been discussed including their molecular pathways involved in stress tolerance. Besides, the review also emphasizes the importance of continued research and implementation of PGPR-based strategies to address the pressing challenges facing global food security including commercialization of PGPR-based bio-formulations for sustainable agricultural.

## 1. Introduction

Besides environmental pressures that plants encounter (biotic or abiotic), they also suffer significant consequences due to their ability to travel from one location to another compared to other living things. As it is well documented, plants often suffer from numerous stressors (biotic and abiotic) during their life cycle. Each one of them can considerably impede plants’ growth and development. The main factor contributing to the global decline in agricultural crop yield is biotic stress, which is brought on by harmful microbes like bacteria, fungi, viruses, insects, and nematodes (Murali et al., 2021a; Gowtham et al., 2022; Hamidian et al., 2023; Karimian et al., 2023). In addition, the abiotic stressors that are detrimental to plants include flooding, drought, soil salinity, extreme temperature, extremely high or low light conditions, contamination with organic pollutants and heavy metals, and excessive radiation (Sarker et al., 2021; Murali et al., 2021a; Ahmad et al., 2022). The abiotic stresses adversely affect the physiological, biochemical, and molecular responses of plants in a multitude of manners, which lowers productivity (Murali et al., 2021a; Munir et al., 2022). The plants grow poorly when exposed to these stresses due to osmotic stress, oxidative stress, reactive oxygen species (ROS) production, hormonal imbalance, ionic toxicity, and reduced nutrient mobilization (Ma et al., 2020).

The abiotic stresses impact plant responses, including the alteration of genes involved in the central metabolic pathways and a change in the growth rate leading to a significant loss in the yield of the crops (Ullah et al., 2021). Most stressed plants point to environmental changes; their roots are where they first respond to such challenging circumstances. Out of the available arable lands, 90% are vulnerable to these stressors, and it is noted that the crop output is reduced and limited to up to 70% when exposed to these abiotic stressors continuously (Waqas et al., 2019). Due to global climate change, drought and soil salinity are two major environmental factors that reduce plant growth and productivity in many plant species, especially in arid and semi-arid regions of the world. The world will face a significant challenge of 70% more food production to adequately sustain the projected 2.3 billion more people by 2050. Therefore, it is imperative to induce stress resilience in crops against drought and salinity stress to meet future generations’ food demands. The plant growth-promoting rhizobacteria (PGPR) have emerged as promising allies in sustainable agriculture, offering the potential to enhance plant tolerance to abiotic stresses, such as drought and salinity. From the literature it has been well noted that these PGPR have been found as effective biological agents in the management of crop plants against drought and salt stress through multi-omic strategies thereby by improving plant growth and production (Kim et al., 2014; Singh et al., 2017; Khan et al., 2021a,b; Mellidou et al., 2021; Vafa et al., 2021; Nishu et al., 2022; Zhao et al., 2022; Patel et al., 2023). The review aims to delve into the multifaceted realm of PGPR-mediated plant resilience, with three primary objectives: (i) to assess the impact of PGPR on enhancing plant tolerance to abiotic stresses such as drought and salt; (ii) to highlight recent advancements in understanding the mechanisms by which PGPR mediate plant resilience; and (iii) to critically evaluate the potential applications of PGPR-based strategies in sustainable agriculture. Previous salient reviews have primarily focused on either general PGPR-plant interactions or specific stress responses but often lack a comprehensive analysis of recent advancements and their implications for sustainable agriculture. Hence, the current study bridges this gap by amalgamating recent evidence to provide a holistic understanding of the impact of PGPR on plant tolerance to drought and salinity, elucidating the latest mechanistic insights, and critically evaluating their potential for sustainable agriculture. Consequently, it aims to offer a comprehensive reference for researchers, agronomists, and policymakers seeking innovative solutions to enhance crop resilience in a changing world.

## 2. Interactive effects of drought and salt stress

The repercussions of climate change have been growing, which has resulted in a sharp rise in drought in recent years. In addition to being an issue, soil salinity is also a result of global warming, especially for crops that require sufficient irrigation (Khan, 2022). Some farmlands are under-irrigated, resulting in salt accumulation inside the soil due to insufficient water supply in many areas. In this situation, the irrigation water either benefits the plants by being utilized or evaporates, leaving salt in the soil. Furthermore, the most salt-affected grounds are found in the arid and semi-arid regions of the world. The main barrier to plant growth is the high salt levels brought on by irrigation, which worsens drought impacts (Ullah et al., 2021). Soil salinity is measured by its electrical conductivity (EC), expressed in dS/m. At the same time, drought is described in the percent (%) field capacity of soil moisture content which can be defined as the amount of water available in the soil. Excessive salt levels are currently thought to have a detrimental impact on 20% of the world’s agricultural land used to cultivate irrigated crops (Khan, 2022). Drought and salinity stress significantly impact future agricultural production, which frequently co-occurs due to changes in climate and the struggle for water, land, and energy (Morari et al., 2015; Mitra et al., 2021; Khan, 2022). Drought and salt stress severely inhibit food crop growth and physiochemical activity. For instance, plants under these stressors have the same morphological and physiochemical traits. It is observed that the drought stress progress in plants is facilitated via greater salt concentrations because salt-related solutes prevent water uptake impacting the leaf water content (Ahluwalia et al., 2021).

The plants explore common and distinct responses to modify plant growth and adaptation under drought and salinity stress. Most plants initially react similarly to drought and salinity, primarily caused by water deficit within the plant which results in a decreased growth rate. After the plants absorb salt, the sodium ions (Na^+^) are transported to the plant’s shoots via the xylem, eventually accumulating in the leaves and shoots (Ullah et al., 2021). The consensus is that sodium ion buildup in plants is harmful because it competes for binding sites with potassium ions (K^+^) essential for cellular activity (Desoky et al., 2020; Hasanuzzaman et al., 2022). Similarly, ROS are developed within the plants that can subsequently induce lipid peroxidation in plant cell membranes, lead to electrolyte leakage from plant cells, and either augment photorespiration or reduce the transpiration ensuing in a slower photosynthesis rate, thereby having a detrimental effect on the production and quality of the plants (Khan, 2022).

**Figure 1:**
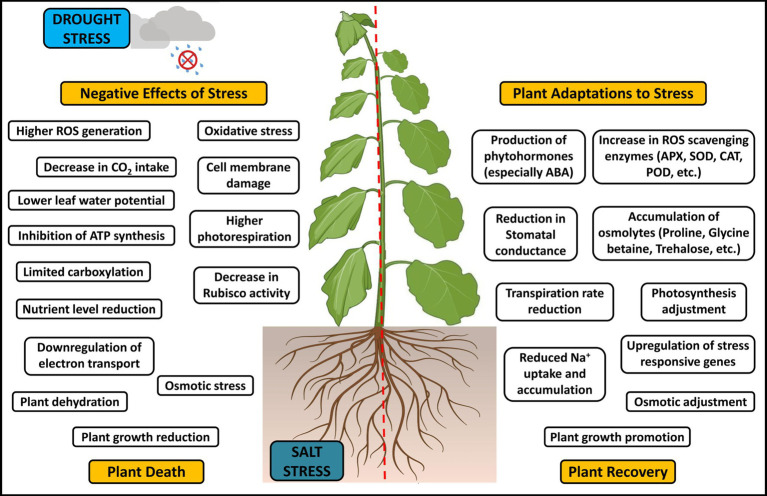
Interactive effects and adaptive mechanism to drought and salt stress in plants.

It is necessary to advance agricultural production in terms of productivity and food safety due to the predicted population growth and rising living standards. Innovative agricultural technologies and production methods are urgently needed to simultaneously achieve sustainable agricultural productivity improvement and environmental and economic sustainability. To overcome salinity stress, plants employ various mechanisms (Figure 1) like (i) control of sodium ion absorption by roots and successive transfer of these ions to leaves, (ii) selective accumulation of sodium ions (Na^+^) in vacuoles or exclusion of these ions, (iii) vacuolar compartmentalization of Na^+^ at the plant cellular level, (iv) modification of plant cell membranes, (v) modulation of the level of plant hormones, and (vi) synthesis of antioxidant enzymes and compatible solutes (Ullah et al., 2021; Khan, 2022). The salt stress tolerance also depends on the cultivars’ growth stage and health, soil composition, microbe association, etc. (Ma et al., 2020). Developing crops that can withstand salt and drought using transgenic technologies and conventional breeding techniques is often expensive, time-consuming, and challenging. Hence, investigating the potent PGPR as a viable replacement for toxic chemicals will help in the improvement of plants’ productivity and soil sustainability even under unfavorable environmental conditions, thereby providing a better perspective for agriculture (Brijesh Singh et al., 2019; Murali et al., 2021a; Hoseini et al., 2022; Munir et al., 2022; Mawar et al., 2023). Additionally, it will endeavor to comprehend the biochemical, physiological, and genetic pathways that the PGPR mediate, as they are crucial for enhancing plant tolerance to these environmental challenges.

## 3. Plant growth-promoting rhizobacteria

The rhizosphere-resident bacteria, commonly termed PGPR, can induce plant growth through several means, which might be either direct or indirect processes. These rhizobacteria are known as effective disease-fighters, helping agricultural productivity and sustainability (Bhat et al., 2022; Gamalero and Glick, 2022; Gowtham et al., 2022). The PGPR that flourishes in the rhizosphere improves plant growth by various mechanisms, such as nitrogen fixation, production of phytohormones, 1-aminocyclopropane-1-carboxylate (ACC) deaminase (Sagar et al., 2020), exopolysaccharides (EPS; Sayyed et al., 2015, 2019; Ilyas et al., 2020), siderophores (Nithyapriya et al., 2021; Srivastava et al., 2022), antioxidants (Gowtham et al., 2022), osmoprotectants (Ilyas et al., 2020), nutrient uptake (Jabborova et al., 2020, 2022; Deepranjan et al., 2021; Kapadia et al., 2021; Sarkar et al., 2021), and induced systemic resistance (ISR; Reshma et al., 2018; Ali B. et al., 2022; Desai et al., 2023) in stressful conditions. These PGPR can also influence plant metabolism and gene expression directly, as well as the expression of root proteins, root morphology, and root growth (Vacheron et al., 2013; Kalam et al., 2020; Basu et al., 2021; Hamid et al., 2021; Ahmad et al., 2022; Lobhi et al., 2022). Besides, PGPR application exogenously can alter the plant rhizosphere microbial communities in soil, which modulates the host’s capacity for nutrient adsorption and pathogen interaction apart from modifying the ability of the plant to tolerate both biotic and abiotic stressors (Hariprasad et al., 2021; Munir et al., 2022). Therefore, they are possible options for chemical fertilizers in agriculture production, which are mentioned below depending on the host and stress factors.

## 4. Mechanisms induced by PGPR during amelioration of drought and salt stress

**Figure 2:**
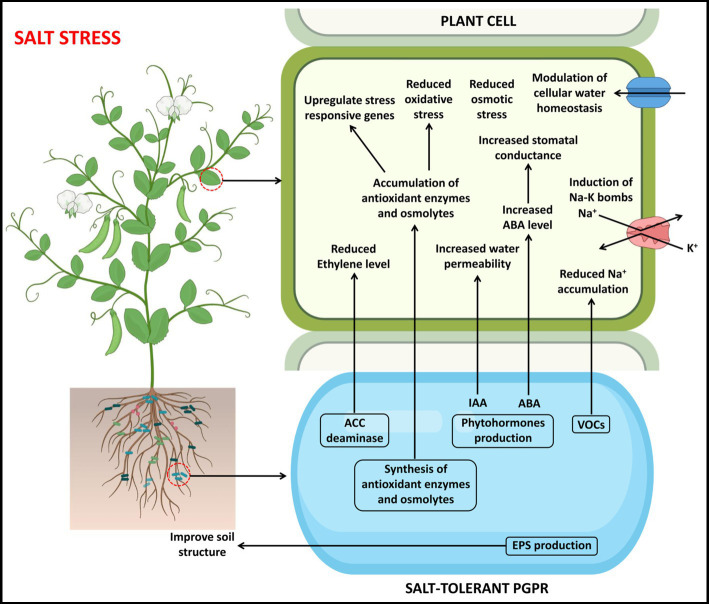
A pictorial overview of the functioning of PGPR in support of plant growth and stress tolerance against drought stress.

**Figure 3:**
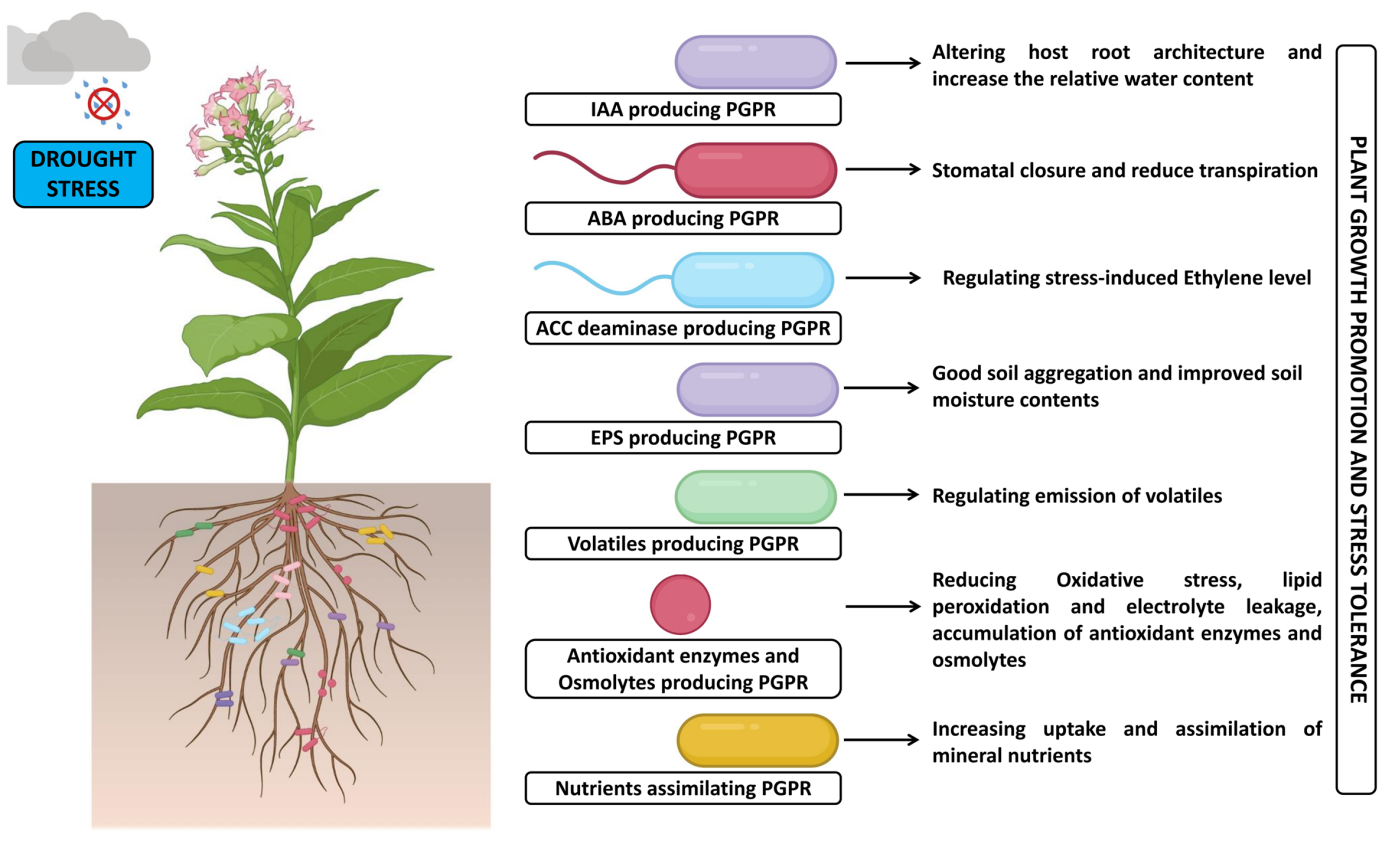
A pictorial overview of the functioning of PGPR in support of plant growth and stress tolerance against salt stress.

The stress-tolerant bacteria can survive better in severe drought and salt stress conditions and overcome their effect by different mechanisms (Fazeli-Nasab and Sayyed, 2019), such as changing plant root system morphology and structure, balancing osmotic stress and oxidative stress, and regulating ion homeostasis (Tables 1, 2). These plausible mechanisms involved during the plant-PGPR interaction to prevail over drought (Figure 2) and salt stress (Figure 3) are discussed below in detail.

**Table 1:** Overview of PGPR-mediated enhancement of plant tolerance against drought stress.

Plants	PGPR Strains/ Consortia	Beneficial traits produced by PGPR	Experimental details	References
*Abelmoschus esculentus*	*Bacillus subtilis*	–	Field experiment	Puthiyottil and Akkara (2021)
*Arabidopsis thaliana*	*Pseudomonas chlororaphis*	Volatiles: 2R,3R-butanediol	Laboratory and pot experiments	Cho et al. (2008)
	*B. endophyticus* and *P. aeruginosa*	IAA, cytokinin, gibberellic aci and EPS	Laboratory experiment	Ghosh et al. (2019)
	*Pseudomonas* sp.	IAA, ABA, gibberellic acid, EPS, and ACC deaminase	Pot experiment	Yasmin et al. (2022)
*Brassica juncea*	*B. marisflavi*	ABA analog/Xanthoxin	Laboratory experiment	Gowtham et al. (2021)
*Cicer arietinum*	*P. putida*	Phosphate solubilization, IAA, and ACC deaminase	Pot experiment	Tiwari et al. (2016)
*Eleusine coracana*	*Variovorax paradoxus, P. palleroniana, P. fluorescens,* and *Ochrobactrum anthropi*	ACC deaminase		Chandra et al. (2020)
*H. annuus*	*P. putida*	EPS		Sandhya et al. (2009)
	*B. subtilis* and *B. thuringiensis*	ACC deaminase		Singh et al. (2019)
*Lactuca sativa*	*B. subtilis*	Cytokinin		Arkhipova et al. (2007)
*Lolium perenne*	*Pseudomonas* sp. and *Bacillus* sp.	ACC deaminase, IAA and EPS	Laboratory and pot experiments	He et al. (2021)
*Medicago sativa*	*B. amyloliquefaciens*	IAA, EPS and siderophores	Pot experiment	Han et al. (2022)
*Mentha piperita*	*P. fluorescens* and *B. amyloliquefaciens*	ACC deaminase and IAA production		Chiappero et al. (2019)
*Mucuna pruriens*	*Enterobacter* sp. and *Bacillus* sp.	IAA and ACC deaminase		Saleem et al. (2018)
		ACC deaminase		Brunetti et al. (2021)
*Ocimum basilicum*	*Azospirillum baldaniorum*	Induced immune response (ISR)	Greenhouse experiment	Mariotti et al. (2021)
*Oryza sativa*	*B. haynesii, B. paralicheniformis* and *B. licheniformis*	ACC deaminase	Pot experiment	Joshi et al. (2020)
	*B. altitudinis* and *B. methylotrophicus*	ABA		Narayanasamy et al. (2020)
	*Gluconacetobacter diazotrophicus*	Nitrogen fixation and IAA	Field experiment	Silva et al. (2020)
*Pennisetum glaucum*	*B. amyloliquefaciens*	ACC deaminase	Pot experiment	Murali et al. (2021a,b)
*Pisum sativum*	Consortia of *Pseudomonas* sp., *O. pseudogrignonense* and *B. subtilis*	ACC deaminase		Saikia et al. (2018)
*Setaria italica*	*P. migulae, P. fluorescens*, and *E. hormaechei*	EPS and ACC deaminase		Niu et al. (2018)
*Solanum lycopersicum*	*Enterobacter* spp.	IAA and gibberellic acid	Laboratory experiment	Bhatt et al. (2015)
	*B. amyloliquefaciens*	EPS	Pot experiment	Wang et al. (2019)
	*Streptomyces* spp.	ACC deaminase and IAA		Abbasi et al. (2020)
	*B. subtilis*	ACC deaminase		Gowtham et al. (2020)
	*Bacillus megaterium*	Extracellular arginine		Morcillo et al. (2021)
*Solanum tuberosum*	*V. paradoxus, P. oryzihabitans* and *Achromobacter xylosoxidans*	IAA and ACC deaminase	Pot and field experiments	Belimov et al. (2015)
	*B. subtilis*	–	Pot experiment	Batool et al. (2020)
*Sorghum bicolor*	*Pseudomonas* sp.	ACC deaminase		Carlson et al. (2019)
	*Streptomyces* sp. and *Nocardiopsis* sp.	Phosphate solubilization, IAA, siderophore, and ACC deaminase		Silambarasan et al. (2022)
*Spinacia oleracea*	*B. amyloliquefaciens* and *Bacillus* sp.	Phosphate solubilization, IAA, and siderophore	Laboratory experiment	Petrillo et al. (2022)
*Trifolium repens*	*B. megaterium and P. putida*	IAA	Pot experiment	Marulanda et al. (2009)
*Trigonella foenum-graecum*	*B. subtilis*	ACC deaminase		Barnawal et al. (2013)
*Triticum aestivum*	*B. thuringiensis*	Reduction in volatile emissions		Timmusk et al. (2014)
	*Klebsiella* sp., *E. ludwigii* and *Flavobacterium* sp.	Phosphate solubilization, EPS, IAA, siderophore, and ACC deaminase		Gontia-Mishra et al. (2016)
	*P. palleroniana* and *P. fluorescens*	ACC deaminase		Chandra et al. (2018)
	*P. stutzeri, Moraxella pluranimalium, E. aerogenes, B. thuringiensis, B. simplex, B. pumilus, B. muralis*, and *B. amyloliquefaciens*	IAA		Raheem et al. (2018)
	*O. anthropi, P. palleroniana*, *P. fluorescens,* and *V. paradoxus*	ACC deaminase		Chandra et al. (2019)
	*Bacillus* sp. and *Enterobacter* sp.	IAA and salicylic acid		Jochum et al. (2019)
	*B. cereus* and *Planomicrobium chinense*	EPS	Field experiment	Khan and Bano (2019)
	*B. subtilis* and *A. brasilense*	EPS, Sugar and Proline	Pot experiment	Ilyas et al. (2020)
	*Streptomyces pactum*	–	Laboratory experiment	Li et al. (2020)
	*B. subtilis*	ACC deaminase	Pot experiment	Sood et al. (2020)
	*P. azotoformans*	EPS		Ansari et al. (2021)
	*P. helmanticensis* and *P. baetica*	Phosphate solubilization, IAA and siderophore		Karimzadeh et al. (2021)
	*Pseudomonas* sp. and *Serratia marcescens*	Phosphate solubilization, ACC deaminase, IAA, siderophore and EPS		Khan and Singh (2021)
	*Chryseobacterium* sp., *Acinetobacter* sp. and *Klebsiella* sp.	IAA and EPS	Jar experiment	Latif et al. (2022)
	*B. megaterium* and *B. licheniformis*	ACC deaminase and IAA	Pot experiment	Rashid et al. (2022)
*Vigna mungo*	Consortia of *Pseudomonas* sp., *O. pseudogrignonense* and *B. subtilis*	ACC deaminase		Saikia et al. (2018)
*Vigna radiata*	*P. aeruginosa*	IAA production	Lab, pot, and field experiments	Uzma et al. (2022)
*Vitis vinifera*	*B. licheniformis* and *P. fluorescens*	ABA	Laboratory experiment	Salomon et al. (2014)
	*P. corrugata* and *E. soli*	ACC deaminase	Pot experiment	Duan et al. (2021)
	*Enterobacter* sp. and *Bacillus* sp.	IAA and salicylic acid		Jochum et al. (2019)
	*B. velezensis*	ACC deaminase and EPS	Laboratory experiment	Nadeem et al. (2020)
	*P. fluorescens*	ACC deaminase	Field experiment	Zarei et al. (2020)
	*Bacillus* spp.	Nitrogen fixation, phosphate solubilization, and IAA and EPS	Pot experiment	Azeem et al. (2022)
*Ziziphus jujuba*	*P. lini* and *S. plymuthica*	ACC deaminase		Zhang et al. (2020)

**Table 2:** Overview of PGPR-mediated enhancement of plant tolerance against salt stress.

Plants	PGPR strains/Consortia	Beneficial traits produced by PGPR	Experimental details	References
*Arabidopsis thaliana*	*B. amyloliquefaciens*	Spermidine	Laboratory experiment	Chen et al. (2017)
	*P. putida*	–		Chu et al. (2019)
	*Stenotrophomonas maltophilia*	Nitrogen fixation	Laboratory experiment	Alexander et al. (2020)
*Avena sativa*	*Klebsiella* sp.	IAA and ACC deaminase		Sapre et al. (2018)
*Capsicum annuum*	*B. licheniformis, Brevibacterium iodinum* and *Zhihengliuela alba*	ACC deaminase	Pot experiment	Siddikee et al. (2011)
*Cicer arietinum*	*Pantoea dispersa*	ACC deaminase and IAA		Panwar et al. (2016)
*Cucumis sativus*	*B. megaterium, P. fluorescens* and *V. paradoxus*	ACC deaminase, IAA, and siderophore	Laboratory experiment	Nadeem et al. (2016)
*Glycine max*	*Bacillus* sp. and *Pseudomonas* sp.	ACC deaminase, IAA, and EPS		Kumari et al. (2015)
*Lactuca sativa*	*Lactobacillus* sp., *P. putida* and *Azotobacter chroococcum*	IAA	Laboratory experiment	Hussein and Joo (2018)
*Medicago sativa*	*Halomonas maura* and *Ensifer meliloti*	EPS	Greenhouse and field experiments	Martínez et al. (2015)
*Mentha arvensis*	*B. pumilus, Exiguobacterium oxidotolerans* and *Halomonas desiderata*	Phosphate solubilization, siderophore, EPS, and ACC deaminase	Pot and glass house experiments	Bharti et al. (2014)
*Oryza sativa*	*Enterobacter* sp.	ACC deaminase, IAA, siderophore, and phosphate solubilization	Laboratory experiment	Sarkar et al. (2018)
	*Glutamicibacter* sp.	IAA and ACC deaminase	Pot experiment	Ji et al. (2020)
	*B. aryabhattai* and *B. tequilensis*	EPS	Glasshouse experiment	Shultana et al. (2020)
	*B. aryabhattai, A. denitrificans* and *O. intermedium*	IAA, phosphate solubilization, and Nitrogen fixation	Pot experiment	Sultana et al. (2020)
	*B. pumilus*	Phosphate solubilization, ACC deaminase, IAA, and EPS		Kumar et al. (2021)
*Pistacia vera*	*Arthrobacter endophyticus, Staphylococcus sciuri* and *Zobellella denitrificans*	ACC deaminase, auxin, siderophore, EPS, and phosphate solubilization		Khalilpour et al. (2021)
*Pisum sativum*	*A. protophormiae*	ACC deaminase		Barnawal et al. (2014)
	*V. paradoxus*	ACC deaminase		Wang et al. (2016)
*Raphanus sativus*	*A. chroococcum, Lactobacillus* sp., and *P. putida*	IAA	Laboratory experiment	Hussein and Joo (2018)
*Solanum lycopersicum*	*Pseudomonas* sp., *Pantoea* sp., *Leifsonia* sp., *Bacillus* sp. and *Arthrobacter* sp.	IAA, siderophore, and phosphate solubilization	Pot experiment	Cordero et al. (2018)
	*Leclercia adecarboxylata*	ACC deaminase and IAA		Kang et al. (2019)
	*Pseudomonas* sp.	ACC deaminase and trehalose		Orozco-Mosqueda et al. (2019)
*Triticum aestivum*	*Bacillus* sp., *B. insolitus* and *Aeromonas hydrophila/caviae*	EPS		Ashraf et al. (2004)
	*Streptomyces* sp.	IAA and siderophore	Greenhouse experiment	Sadeghi et al. (2012)
	*E. cloacae, P. putida, P. fluorescens* and *S. ficaria*	ACC deaminase	Field experiment	Nadeem et al. (2013)
	*Klebsiella* sp.	ACC deaminase	Pot experiment	Singh et al. (2015)
	*B. subtilis* and *Marinobacter lipolyticus*	EPS		Talebi Atouei et al. (2019)
	*P. fluorescence, E. aurantiacum* and *B. pumilus*	Phosphate solubilization, ACC deaminase, and IAA		Nawaz et al. (2020)
	*B. amyloliquefaciens*	Spermidine	Laboratory experiment	Chen et al. (2017)
	*B. aquimaris*	IAA		Li and Jiang (2017)
	*B. subtilis* and *B. safensis*	ACC deaminase	Pot experiment	Misra and Chauhan (2020)
	*E. cloacae*	Siderophore, IAA, and EPS ACC deaminase		Ali S. A. M. et al. (2022)

### 4.1. Changes in plant root system architecture

The root system architecture describes the entire spatial configuration and covers the root’s density, angle, surface area, volume, and biomass. Plants with a larger root architecture help in higher water absorption from the soil, thereby leading to the modification of root morphology that allows them to cope under drought stress and variations in root morphology are found to be species-specific (Paez-Garcia et al., 2015). Inoculating agricultural crop plants with PGPR can lead to significant changes in the root system architecture, wherein the changes are observed through stimulation of root elongation and density which helps in nutrient and water uptake. Besides, PGPR is known to induce the formation of lateral roots and root hairs that assist in nutrient absorption, stabilizing the plant in the soil, promote deeper root growth, enhance the formation of mycorrhizal associations for symbiotic relationships with plant roots, fix atmospheric nitrogen, and support stress tolerance. The changes in root system architecture induced by PGPR ultimately contribute to improved plant growth, increased crop yield, and better crop health (Grover et al., 2021; Mohanty et al., 2021; Gowtham et al., 2022).

The rhizosphere microbial communities significantly influence host plant health and phenotypic traits by changing the soil processes in stressful conditions (Grover et al., 2021). During water stress conditions, some bacteria alter the cell membrane elasticity in root cells; these modifications are considered the first step in improving drought tolerance (Vacheron et al., 2013). Mishra et al. (2020) have noted that inoculating *Zea mays* with *Ochrobactrum* sp. under drought conditions improved the development of root hairs, root length, and dry weight. The combination and concentration of mineral nutrients in the soil substantially influence plant growth and development. The plants can retain enough nutrient content despite shifting soil environments through changes to root architecture, the emergence of root-based transport systems, and symbiotic relationships with helpful soil bacteria (Naylor and Coleman-Derr, 2017). It is important to note that the specific changes in root system architecture can vary depending on the crop species, the strain of PGPR used, and environmental conditions (Brijesh Singh et al., 2019; Murali et al., 2021a; Gowtham et al., 2022). Therefore, selecting appropriate PGPR strains and implementing proper agronomic practices are essential for maximizing the benefits of PGPR inoculation in agriculture. As a result, it was discovered that PGPR strains may enhance soil fertility, control pH, safeguard crops from phytopathogens, and lessen the effects of abiotic stress on various crops.

### 4.2. Balancing osmotic stress

Osmotic stress is the first immediate effect caused by drought and salt stress, which disrupts leaf water potential (Ψ) and causes stomatal closure, generates ROS, membrane lipid peroxidation, and increases antioxidant enzymatic activities and accumulation of osmolytes in plants (Brijesh Singh et al., 2019; Gowtham et al., 2022). In contrast, the stomatal limitations reduced the efficiency of photosystem II and limited CO_2_ assimilating enzyme activities, which are the significant challenges posed by the plants that lead to reduced photosynthetic rates under extreme drought conditions (Batool et al., 2020). Due to the imbalanced gas exchange and decreased leaf area, photosynthesis slows down, and therefore, to mitigate the effect of these stressors on plants, they should sustain water homeostasis and maintain their photosynthetic structures unharmed. Further, the osmotic stress brought on by salt and drought directly impacts numerous soil processes, including stressing out the microorganisms (Hasanuzzaman et al., 2022). When under stressful conditions, the soil bacteria adjust their osmotic conditions and sustain themselves hydrated by cellular compatible solute accumulation that assists in maintaining the right amount of water in their cells (Desoky et al., 2020).

Meenakshi et al. (2019) and Abbasi et al. (2020) have illustrated that *Solanum lycopersicum* and *Triticum aestivum* plants’ vulnerability to the adverse effect of drought stress decreased the bacterial inoculation that assisted in the increased water usage effectiveness, maintaining cell membrane integrity and RWC in the infected plants’ shoot and root tissues. In addition, *Setaria italica* plants showed better growth under drought conditions due to treatment with *Pseudomonas fluorescens*, which increased soil moisture by colonizing both the root surface and the soil adhering to them (Niu et al., 2018). Besides, using *Bacillus subtilis,* a PGPR strain, enhanced the RWC in tomato plants and improved plant growth compared to untreated plants (Gowtham et al., 2020).

The plant-rhizobacterial interactions are researched to date to understand better the mechanisms implicated in PGPR-mediated osmotic stress tolerance. These bacterial associations significantly impact the formation of extracellular chemicals, increased food availability in the rhizosphere, and protection against abiotic challenges affecting plant growth. Accordingly, exopolysaccharides (EPS), volatile organic compounds (VOCs), and suitable osmolytes, which are produced by bacteria outside of their cells, operate as signal molecules for plant growth in challenging environments (Ma et al., 2020; Kapadia et al., 2022; Khumairah et al., 2022; Sagar et al., 2022a). The EPS are highly hydrated organic polymers with fundamental defensive roles against abiotic stress during plant-microbe interactions (Sayyed et al., 2015; Ahmad et al., 2022). The EPS production is assumed to be directly responsible for the regulation of water potential, aggregation of soil particles, ensures requisite communication between rhizobacteria and plant roots, resulting in the sustainability of the host under initial osmatic stress (Naseem et al., 2018; Ilyas et al., 2020).

Similarly, PGPR has been employed to successfully combat the severe impact of drought through uplifting the EPS production and also through rhizome-sheaths formation around the roots to guard against dehydration, thereby denoting the importance of EPS-producing PGPR in reducing the scarcity of water and improving global food security (Ahmad et al., 2022). The EPS-producing PGPR, including *Agrobacterium* spp., *A. vinelandii*, *Rhizobium* sp., *R. leguminosaru*, *Bacillus drentensis*, and *Xanthomonas* sp., are synergistically essential for nourishing the soil and supporting crop production under salinity stress (Mahmood et al., 2016). Likewise, Ma et al. (2020) have confirmed that the EPS-producing PGPR maintained soil aggregation, dehydration, and water potential, which are critical in improving nutrient uptake that directly correlates to enhancing plant growth. Nevertheless, relief of osmatic stress in plants mainly relies on the growth stage, intensity, and period of the stress and the efficiency of PGPR application to relieve osmotic stress. Therefore, it is important to identify the rhizobacteria that produce EPS to get over the adverse effect of drought and salt stress in crop plants.

The VOCs are a mixture of low molecular weight volatile compounds produced by bacteria that possess antibacterial properties and other cross-talk interactions with the plant pathogens and their host (Tilocca et al., 2020). These bacterial volatiles can alter the formation or dispersal of biofilms and alter bacterial motility, including ammonia, nitric oxide, hydrogen sulfide, trimethylamine, and 2-amino-acetophenone (Schulz-Bohm et al., 2017). Some bacteria produce VOCs, which can change how other bacteria behave, control the level of antibiotic resistance, and have antagonistic effects on other nearby bacteria in the rhizosphere. The emission of VOCs plays an intriguing signaling function during the association of microbes, and it is well observed that certain rhizobacterial species can secrete or emit VOCs as extracellular molecules, which can alleviate the osmotic stress originating due to drought and salt stress (Russo et al., 2022). Similarly, Vaishnav et al. (2015) have noted an improvement in the production of VOCs upon inoculation with the PGPR *Pseudomonas simiae* strain, which in turn improved the growth, enhanced proline and chlorophyll content in *Glycine max* seedlings and elicited the induced systemic tolerance against osmotic stress caused by salt stress condition. The microbial VOCs (acetoin being the main one) produced by *Bacillus amyloliquefaciens* were able to significantly alter the morphological characters that helped in the higher accumulation of total chlorophyll and also helped in the reduction in the ABA levels in *Mentha piperita* under salt stress when compared to uninoculated plants (Cappellari and Banchio, 2020). The VOCs produced from PGPR strains are the factors for triggering induced systemic tolerance in the plant against stressors. Similarly, the better exploitation of VOCs emitted from these rhizobacteria has been noted as the prime plant defense mechanism for regulating their growth and enhancing stress tolerance through interactions with the phytohormones (Sudha et al., 2022).

**Figure 4:**
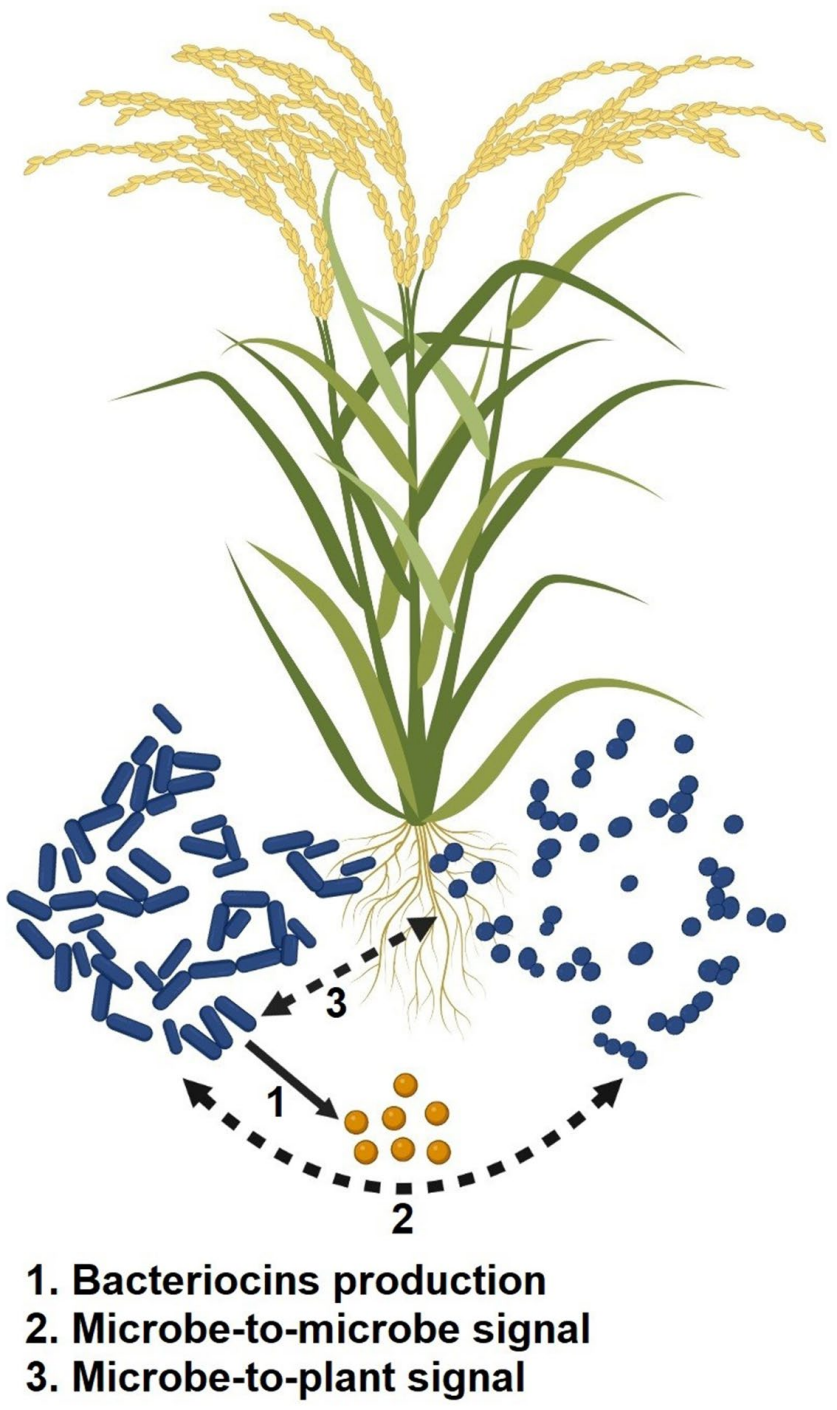
Mechanism of action of bacteriocins excreted by PGPR in abiotic stress resilience.

To lessen competition for nutrient resources and niche spaces, various bacteria excrete antimicrobial compounds (such as bacteriocins; Subramanian and Smith, 2015). The most numerous and varied class of the bacteria’s defense systems are the bacteriocins, which are antimicrobial peptides produced by ribosomal enzymes. The development of fruitful plant-microbe partnerships is controlled by a large number of bacteriocin-producing PGPR strains, which create a variety of bacteriocins and exchange them in the rhizosphere (Nazari and Smith, 2020; Shah et al., 2021). In the rhizosphere, the bacteriocins function as signaling molecules between microbes or between microbes and plants (Figure 4). When exposed to abiotic stress, it not only prevents rival microorganisms from occupying its niche but also physically widens the niche by enhancing plant growth, thereby acting as a biostimulant agent for the sustainable agricultural industry.

The maintenance of osmotic equilibrium and to have a better response to drought and salt stress, plants lose intracellular water, which leads them to amass appropriate osmolytes in the cytoplasm which include trehalose, proline, etc. (Gowtham et al., 2022). The PGPR increases the amassing of osmolytes in the host plant as a defense mechanism against osmotic stress in environmental stressors. Besides, these microbes manufacture osmolytes faster than the plants linked with them. The defense mechanism the PGPR uses to counteract the osmotic stress in environmental stresses involves the enhanced accumulation of osmolytes in the host plant. Moreover, osmolytes are more quickly produced by the PGPR than their associated plants as their inoculation in plants improves the production of osmolytes, which may be related to the roots absorbing bacterial solutes or *de novo* synthesis in plants (Ma et al., 2020). When exposed to salinity, osmolytes produced by PGPR can improve root hydraulic conductivity and water potential, which benefits the plant’s stomatal opening and transpiration rate (Ilangumaran and Smith, 2017). Therefore, the accumulation of certain osmolytes has facilitated various plants to resist drought and salt stress in beneficial PGPR.

One of the compatible solutes a plant makes when its water supply is cut off is proline. It decreases the cells’ water potential and helps sustain turgor pressure, assuring the plant’s development, metabolism, and yield rate. Proline content is closely correlated with drought stress, and proline concentration is highly linked with stress intensity. Due to water constraints, cells with a high proline concentration maintain their water balance and membrane stability (Khan et al., 2021a,b). Therefore, evaluating the proline content is essential to determining how plants are sensitive and resistant to abiotic stressors. Among the several osmolytes, the increase of proline content within the bacteria amid osmotic stress has drawn much attention and the PGPR can also control how proline is expressed in plants. Glycine betaine is one of the well-known compatible osmolytes which help increase resistance to abiotic stresses (specifically drought and salt) by improving water status and protecting cell membranes from ROS (Khan et al., 2020; Sagar et al., 2020; Kusale et al., 2021a). In addition, glycine betaine has been well documented to augment the plant defense mechanisms, including the osmotic balance, enzyme activities, and genes associated with the tolerance. The plants exposed to PGPR inoculation under drought and salinity conditions exhibited a more significant accumulation of glycine betaine content as a major factor in reduced water loss. The higher glycine betaine content was noticed in drought-stressed *Z. mays* plants inoculated with *Pseudomonas* spp. than in untreated plants (Sandhya et al., 2010). *Pseudomonas putida* and other rhizobia have been reported to mitigate drought stress in wheat (Najafi et al., 2021; Tanvere et al., 2023). Choline is another crucial osmolyte produced from glycine betaine accumulation to cope with drought stress. Studies have demonstrated that the microbial populations in the soil contribute significantly to the buildup of choline, a precursor to the metabolism of glycine betaine (Ghosh et al., 2021).

Trehalose is a non-reducing sugar that protects against extreme abiotic stress, like salt and drought, by stabilizing sugar glasses, vitrifying sugar molecules, and acting as a xeroprotectant in plant and bacterial cells (Ahmad et al., 2023). Trehalose helps plants to communicate because it can keep the osmotic balance in their cells and protect the biological structures from damage during desiccation. The stress tolerance signal pathway can be established by applying even a small quantity of trehalose to the plant roots. Trehalose promotes the survivability of PGPR and other biocontrol agents when they are commercially formulated and stored for longer durations, besides boosting the competence of these microbes in the root regions and rendering a hand in the resistance against abiotic stresses. *Azospirillum brasilense* inoculation of *Z. mays* plants results in the conferment of drought resistance and a significant enhancement of root and leaf biomass (Rodríguez-Salazar et al., 2009).

Polyamines are the low molecular weight aliphatic amines, which play the complex role as an osmolyte associated with plant growth promotion which is subjected to abiotic stress response by accelerating the function of enzymes and genes involved in the antioxidant defensive system and ROS homeostasis (Chen et al., 2019). The three primary polyamines found in plants like, spermine, spermidine, and putrescine, are also crucial in how they react to biotic and abiotic stressors. Exogenous polyamines have been shown to increase drought and salinity tolerance, but further study is needed to comprehend how polyamines released by the PGPR ultimately affect plants. Under osmotic stress, *Oryza sativa* seedlings accumulated polyamines due to the inoculation of *A. brasilense* (Cassán et al., 2009). The spermidine secreted by the beneficial rhizobacterium *Bacillus amyloliquefaciens* reduced the impact of oxidative damage, decreased the toxicity of Na^+^, and ABA accumulation in *Z. mays* was inhibited, thereby resulting in the improvement of plant salt sensitivity (Chen et al., 2017). The regulation of endogenous free polyamines in plants by the PGPR under water and salt stress was found to elevate the antioxidant defense capacity and encourage the expression of genes linked to antioxidants.

### 4.3. Balancing oxidative stress

Oxidative stress is critical for plants due to the large amounts of ROS produced in the membranes, which, under abiotic stress, can result in severe denaturation of protein, DNA mutation, and membrane lipid peroxidation (Chaves and Oliveira, 2004). PGPR employment can suppress the oxidative stress level by balancing the level of phytohormones, maximizing the activities of antioxidants and production of osmoprotectants, and correcting ion imbalance in plants grown under water deficit and salinity (Batool et al., 2020). The phytohormones are endogenous substances with a lower molecular weight that efficiently trigger the immune system’s response to biotic and abiotic stresses. Due to these abiotic stressors, the plants produce many hormones that include indoleacetic acid (IAA), ethylene, and abscisic acid (ABA), which aid the plant’s defense system (Ma et al., 2020). These hormones alter the metabolism, morphology, and other systems of plants. Bacterial hormone production and its ability to activate endogenous hormones are essential for increasing drought tolerance (Singh and Jha, 2017; Jochum et al., 2019). Furthermore, soil bacteria could directly impact plants’ hormonal equilibrium by generating exogenous phytohormones. As a result, it is believed that alterations in hormone signaling, mediated by interactions between plants and microbes, are a likely mechanism for causing plants to tolerate drought and soil salinity (Hariprasad et al., 2021).

One of the main auxins, the IAA is physiologically crucial for the growth and development of plants as it is involved in cell division, structure of xylematic vessels, root branching, root elongation, differentiation of vascular tissues, phototropism, gravitropism, and plant tolerance to adverse environmental conditions. The IAA may have positive benefits when present in the optimum concentrations, but too much of this auxin can harm the plants in adverse environmental conditions. Multiple studies have shown that exogenous IAA typically applied to plants decreased drought and salt stress by controlling the photosynthetic rate, the effectiveness of water usage, and Na^+^ buildup (Desoky et al., 2020). The higher auxin level may help maintain plant growth, especially roots and leaves, as these are the significant elements that serve in their better resistance under an abiotic stress environment. The IAA production from soil bacteria is widespread and originates from many taxonomic groups (Gowtham et al., 2017).

Some PGPR produces IAA by converting L-tryptophan using several biosynthetic pathways (Ahmad et al., 2020). These pathways involve intermediates such as indole-3-acetamide (IAM), indole-3-acetaldoxime (IAOx), indole-3-pyruvic acid (IPyA), and tryptamine (TAM). The four biosynthetic pathways leading to IAA production from L-tryptophan in bacteria include (i) IAM pathway wherein L-tryptophan is first converted into indole-3-acetamide (IAM) through the action of the enzyme tryptophan decarboxylase which gets transformed into IAA by the action of amidase enzymes; (ii) IAOx pathway in which L-tryptophan is first converted into IAOx through the action of tryptophan-2-monooxygenase and subsequently into IAA by the action of the enzyme indole-3-acetaldoxime hydrolase; (iii) IPyA pathway where L-tryptophan is first converted into IPyA through the action of tryptophan aminotransferase and then converted into IAA by the action of the enzyme indole-3-pyruvate decarboxylase and (iv) TAM pathway that includes conversion of L-tryptophan into TAM primarily through the action of tryptophan decarboxylase and subsequently into IAA by the action of the enzyme tryptamine 5-hydroxylase. The pathways mentioned above may be found in various bacteria that can produce IAA from L-tryptophan to impact plant growth promotion. However, the tryptophan-independent pathway for IAA production has been described in *Azospirillum brasilense,* wherein the bacterium could produce IAA without relying on an exogenous tryptophan supply (Prinsen et al., 1993). In addition, the enzymes that are participatory in the tryptophan-independent pathway have not been identified yet. It is important to note that not all bacteria can synthesize IAA, irrespective of the pathways identified. Plant roots may uptake IAA synthesized by bacteria, thereby increasing the inherent plant pool. Enhanced IAA generally stimulates plant growth, suppressed when their accumulation is elevated. The PGPR-producing IAA improves root system architecture, increases water permeability into cells, increases leaf uptake, and regulates metabolic homeostasis to mediate abiotic stress tolerance. Moreover, IAA-producing PGPR is found to alleviate the agricultural production losses that result due to drought and salinity stress.

Commonly referred to as a “plant stress hormone” ethylene plays a part in several biological processes in plants, including the ripening of fruits, flowering, seed germination, leaf abscission, and tissue differentiation, and also manages elongation and branching of roots (Shekhawat et al., 2023). However, under biotic and abiotic stressors, a substantial amount of ethylene is produced within the plants, which limits the growth of the root, shoot, and leaf, resulting in plant growth restriction (Brijesh Singh et al., 2019; Murali et al., 2021a). The precursor molecule of ethylene, 1-aminocyclopropane-1-carboxylate (ACC), is produced by involving ACC synthase as the first step of its synthesis, and ACC oxidase then transforms ACC into ethylene. The rhizobacteria can considerably produce ACC deaminase, which converts ACC into α-ketobutyrate and ammonia. By preventing the production of ACC and ethylene, the PGPR impacts the plant’s ethylene cycle (Gowtham et al., 2020; Murali et al., 2021b). Thus, the levels of ethylene inside plants did not rise to the levels detrimental to plant growth. The scientific literature showed the effectiveness of PGPR in producing ACC deaminase enzyme, which can significantly promote plant development and enhance plants’ ability to tolerate ethylene generated under salt or drought stress by lowering the ACC produced by the plants and maintaining the same at appropriate levels. It is well known that the bacterial-produced ACC deaminase is linked to the ability of many crops to withstand salt and drought stress (Chandra et al., 2019; Danish et al., 2020).

By reducing levels of stress-induced ethylene and minimizing related growth inhibition, the PGPR containing ACC deaminase may be able to lessen the impacts of stress and increase plant growth under these conditions. Similarly, *S. lycopersicum* and *Z. mays* treated with ACC deaminase-producing *B. subtilis* and *Achromobacter xylosoxidans*, respectively, were able to protect the plants from drought-induced oxidative damage by regulating plant ethylene levels (Danish et al., 2020; Gowtham et al., 2020; Sagar et al., 2022b). Moreover, some PGPR are known for their mutual production of IAA and ACC deaminase under adverse environmental conditions by promotion of cell division and root growth (by IAA) and hydrolyzation of excess amount of ACC and ethylene (by ACC deaminase) apart from improving the plant growth. The synergistic effects of bacterial IAA and ACC deaminase will help the plant to withstand adverse environmental conditions. However, the additional PGPR plant-beneficial characteristics, including the production of IAA and osmoprotectant molecules, are closely attributed to bacterial ACC deaminase activity (Sagar et al., 2020).

The ABA is a stress-related phytohormone primarily produced to defend plants against drought and salt stress. Under stress, the ABA can be transported from the roots to the leaves. Numerous PGPR act as plant ABA content modulators and can alter ABA levels in plants, allowing for the regulation of salt and drought stress. According to studies, PGPR treatments raised the levels of ABA in plants. According to Cohen et al. (2009), ABA-producing *Azospirillum lipoferum* strains can still protect *Z. mays* plants from the osmotic damage caused by drought stress. According to Salomon et al. (2014), ABA-producing *Pseudomonas* and *Bacillus* strains operate as stress relievers and aid *Vitis vinifera* plants in dealing with drought stress by promoting ABA production and thereby reducing the rate of plant water loss. In this regard, the evidence generally suggests that the PGPR capable of producing ABA is considerably utilized for abiotic stress management in plants.

The PGPR not only produce phytohormones but also fix nitrogen, sequester iron-chelators (siderophores), and solubilize phosphate for the plants, enhancing their capacity to absorb soil nutrients and reduce the adverse effects of salt stress and drought (Raheem et al., 2018; Gontia-Mishra et al., 2020). The Dissimilatory Nitrate Reduction to Ammonium (DNRA) is a microbial process (anaerobic respiration) that involves the reduction of nitrate (NO_3_^−^) to ammonium (NH_4_^+^), and the process occurs in certain bacteria, including some saprophytic bacteria like *Bacillus* and others (Sun et al., 2016; Liu et al., 2021). The saprophytic bacterium takes nitrate (a common form of nitrogen available) from their environment and is enzymatically reduced to nitrite (NO_2_^−^) inside the bacterial cells. Further reduction of nitrite occurs, leading to the formation of intermediates like nitric oxide (NO) and eventually nitrous oxide (N_2_O) is an essential step in the DNRA process. The nitric oxide (NO) and/ or nitrous oxide (N_2_O) are further enzymatically reduced to ammonium (NH_4_^+^) which can be used as a nitrogen source for the growth and metabolism of the bacteria (Sun et al., 2016). Overall, the DNRA process helps certain saprophytic bacteria to obtain energy by using nitrate as a terminal electron acceptor under anaerobic conditions and plays a significant role in the cycling of nitrogen compounds and affects the availability of nitrogen to the host plant. It is important to note that not all bacteria can perform DNRA, as it depends on their specific metabolic pathways and the presence of relevant enzymes. The DNRA activities are prevalent in many bacteria, such as *Shewanella loihica*, *S. oneidensis,* and *S. putrefaciens,* which harbor competing dissimilatory nitrate reduction pathway with the periplasmic nitrate reductase (Nap) genes (*nrfA, nirS,* and *nirK*) required for the first step of nitrate reduction (Nojiri et al., 2020; Liu et al., 2021). The rhizobacterial strains which accomplish the dissimilatory nitrate ammonification exhibit the sequential reduction of nitrate to nitrite and subsequently to ammonium under anaerobic conditions. Therefore, the relative contribution of DNRA activities of rhizobacteria plays an important role in plant growth, nitrogen balance, and even climate change.

The availability of essential nutrients, including nitrogen, is necessary for plant growth and productivity. The nitrogen-fixing bacteria are crucial for biological nitrogen fixation under abiotic stresses (Jabborova et al., 2021). An enzyme nitrogenase complex present in bacteria is responsible for the nitrogen-fixation mechanism. These bacteria have the regulation of nitrogenase genes, which are necessary for nitrogen fixation as well as the synthesis and regulation of enzymes. The halo-tolerant PGPR possessing the potential to absorb the nitrogen will considerably improve the K^+^/ Na^+^ ratio by inhibiting Na^+^ uptake and elevating K^+^ and Ca^2+^ in salt-sensitive plants such as *Glycine max* and *Triticum aestivum* (Desoky et al., 2020; Hasanuzzaman et al., 2022). The PGPR controls the exchange of micro- and macro-nutrients, which decreases the buildup of Na^+^ and Cl^−^ ions. The use of specific bacteria is to enhance nutrient availability and improve the nutrient content of plants, particularly in terms of zinc uptake and accumulation in the rhizosphere. In this context, certain bacteria, especially zinc-solubilizing and zinc-accumulating bacteria, play a crucial role in increasing zinc availability and uptake by plants (Yadav et al., 2022). Iron deficiency is the primary constraint inducing plant chlorosis, eventually impacting crop quality and productivity (Han et al., 2022). Siderophores are the small organic compounds produced by some gramineous plants and microbes in iron-deficient environments, allowing plants to absorb iron from their surroundings even when there is less iron (Saha et al., 2016). Under unfavorable conditions, the siderophores are crucial for phytostabilization, offer metal coalescence, enhance plant development, and lower soil metal bioavailability. The pathogen is depleted of essential iron due to the formation of siderophores that firmly attach to soil Fe^3+^. By increasing the amount of iron in the environment, PGPR like *Azotobacter vinelandii*, *Bacillus* spp., and *Pseudomonas* sp. use siderophores produced to fulfill their requisite iron requirements in the rhizosphere (Ferreira et al., 2019). It was observed that the siderophores produced by PGPR are gaining more attention due to their function as iron chelators and their advantage over the application of synthetic chelating agents in terms of biodegradability. The usage of siderophores is practically limited in agriculture due to their complex structure and difficulty in production with low yield.

Under abiotic conditions like drought, salt, etc., the plants typically experience a nutritional shortfall due to a lack of phosphorus, which is primarily found in the soil in both organic and inorganic forms (Bechtaoui et al., 2021). Plants’ insoluble phosphorus contributes to the phosphorous deficit, yet plants can only take up phosphorous as monobasic and dibasic ions. Plants may benefit from the phosphorus-solubilizing bacteria that can assist them with water shortages and overcome the limited phosphorus availability to plant systems in rhizospheric soil (Kour et al., 2020). Phosphate-solubilizing bacteria like *Serratia, Azotobacter, Bacillus, Burkholderia, Enterobacter, Erwinia, Flavbacterium, Microbacterium,* and *Rhizobium* can be employed as biofertilizers. Rhizobacteria can solubilize the inorganic phosphorus that the plants cannot absorb, aiding plant development (Munir et al., 2022). The improvement of agricultural production is achieved by PGPR-mediated phosphate-dependent regulation under abiotic stress conditions, while the phosphorus solubilization might be attributed to the synthesis of organic acids by the PGPR at the rhizosphere (Kour et al., 2019).

The overproduction of ROS typically brought on by drought and salt stress damages normal cell metabolism by causing oxidative damage to DNA, lipids, and membrane proteins (Hasanuzzaman et al., 2022). Malondialdehyde (MDA), a lipid peroxidation marker that also serves as an oxidative stress marker, is frequently referred to as MDA. According to earlier research, beneficial bacteria can help plants develop under drought stress by lowering MDA levels, avoiding ROS buildup, and stimulating antioxidant enzyme activities (Abdelaal et al., 2021; Murali et al., 2021b). Plants use the principal enzymatic ROS scavenging system to reduce high ROS levels to protect themselves from oxidative stress. Salt-stressed *Glycine max* plants have higher levels of antioxidant enzymes (such as GSH and SOD) after being inoculated with halotolerant rhizobacterial strains (Khan A. et al., 2019; Hasanuzzaman et al., 2022). Soil bacteria activate the antioxidant system to improve cell membrane stability, increasing drought resistance. The PGPR regulates the antioxidant enzyme activity to prevent oxidative damage due to drought stress. The expression of antioxidant genes was increased after PGPR application, which enhanced the activities of antioxidant enzymes. Superoxide was reduced by the increased enzyme activity, which also shielded the chloroplast from ROS impact. According to Zhang et al. (2020), ACC-deaminase-producing bacterial-treated *Ziziphus jujuba* plants significantly reduced the MDA level by enhancing the activity of antioxidant enzymes (POD and SOD) compared to non-inoculated plants with increased water stress. ACC deaminase-producing *B. subtilis*-treated plants can boost APX and SOD activity by lowering MDA and H_2_O_2_ contents compared to control plants cultivated in extreme drought conditions (Gowtham et al., 2020). Accordingly, it can be deduced from the literature that PGPR treatment boosted enzyme activities and decreased the MDA level under water stress by increasing the plant’s capacity for scavenging and controlling the expression of antioxidant genes.

Gene expression research can be used to compare and comprehend how an organism responds to its surroundings. Recent studies using molecular methods have examined how genes are expressed under drought stress in relation to PGPR-induced tolerance (Ghosh et al., 2019; Gowtham et al., 2022). Each PGPR has a unique gene set that enables it to respond in various protective ways to the damaging impacts of abiotic stressors like salinity and drought. A number of PGPR can change a plant’s gene expression, increasing the output of stress-protective substances such ROS detoxifying enzymes and osmolytes. IAA secretion (*iaaM*), nitrogen fixation (*nifU*), phenazine (*phzCEF*), siderophore (*sbnA*), and spermidine (*speB*) production are among the functional genes found in the PGPR that have been linked to plant growth promotion as well as stress tolerance (Xiong et al., 2019). Khan A. et al. (2019) have shown that the expression of soybean salt tolerance 1 (*GmST1*) and IAA-mediating (*GmLAX3*) genes was found to be upregulated upon the inoculation with the halo tolerant rhizobacterial strains in salt-stressed *Glycine max* plants. The PGPR inoculation can also cause the up-regulation of proteins associated with phosphatase activity related to phosphate solubilization.

### 4.4. Regulation of ion homeostasis

When the influx of ions exceeds the exclusion rate, salinity accumulates hazardous Na + and Cl − concentrations within leaves. Initially, the plants compartmentalize the excess salts in vacuoles to prevent their buildup in the cytosol and intracellular spaces, impeding respiration and photosynthesis. The ability of the soil bacteria to sustain ion homeostasis must be advantageous for plant development and salinity tolerance. By ensuring a high K^+^/Na^+^ ratio, the PGPR can control the homeostasis of hazardous ions. It reduces the buildup of Na^+^ and Cl^−^in leaves, boosts ion exclusion from root cells, activates the development of ion transporters, and controls the exchange of micro- and macro-nutrients (Kusale et al., 2021b). The plasma membrane-bound proteins known as high-affinity K^+^ transporters (HKTs) mediate Na^+^ transportation in plants and prevent excessive Na^+^ ion concentrations from building up in the shoots by preventing them from reaching the roots. The inoculation of rhizobacteria influences the expression of several ion affinity transporters that help maintain cellular ion homeostasis in salt-stressed plants, which requires tissue-specific regulation of the HKT genes during plant-microbe interactions. It has been reported that rhizobacterial inoculation controls the expression of various ion affinity transporters. The tissue-specific regulation of HKT genes is essential in plant-microbe interactions for maintaining cellular ion homeostasis in salt-stressed plants. Salt overload sensitive (SOS) genes and other enzymes that play a role as sodium antiporters can help plants adapt to salt stress. By decreasing the concentration of the ion Na^+^ and increasing the absorption of K^+^ion in salt-stressed *Glycine max* plants, the introduction of halotolerant rhizobacterial strains preserved the osmotic equilibrium (Khan M. A. et al., 2019). Microorganisms produced during EPS synthesis protect plants from harmful ion absorption. They act as a physical barrier that guards against ion toxicity and safeguards the root system. The EPS can attach to cations like Na^+^, making it impossible for plants to absorb in saline surroundings.

## 5. Cry for help

Filed research in plant-microbe interactions has shown that the composition and diversity of the microbial community in the rhizosphere play a crucial role in the plant’s ability to withstand stress. Indeed, the “Cry for Help” concept is a significant aspect of microbial recruitment in the plants’ rhizosphere under abiotic and biotic stresses. During their lifecycle, plants encounter various stress, such as drought, salinity, pathogen attack, or nutrient deficiency, wherein plants release specific chemical signals, known as VOCs, root exudates, or other chemical signals, into the rhizosphere. These exudates serve as a salt overly sensitive signal to attract beneficial microbes that can aid the plant in mitigating the stress and improving overall growth and health. The process of attraction through these chemical signals is called microbial recruitment. Several functions are attributed to the recruited microbes in the rhizosphere, including enhanced nutrient acquisition, pathogen suppression, plant growth promotion, and abiotic stress tolerance. Several key points are essential in understanding the concept of Cry for Help in microbial recruitment in the rhizosphere, like chemical signaling, microbial diversity, mutualistic relationships, enhanced stress tolerance, and induced systemic resistance (ISR). The study of Cry for Help and microbial recruitment in the rhizosphere is essential for understanding the complex interactions between plants and microbes. It has implications for sustainable agriculture and environmental management. By harnessing the power of beneficial microbes, it may be possible to develop strategies to enhance plant resilience to various stresses and reduce the reliance on chemical inputs in agriculture.

## 6. Key challenges and multi-omics approach

Several key challenges need to be addressed before widespread adoption and successful commercialization can be achieved through PGPR by ensuring consistent and reliable results across different crops and environments. Successful integration of PGPR into existing conventional agricultural practices is essential as it is prone to specific challenges, which include (i) the maintenance of viability and activity of the bacteria during storage to achieve desired results in the field; (ii) compatibility with other agricultural inputs, such as fertilizers and pesticides to avoid any negative interactions; (iii) obtaining regulatory approval for the commercial use of PGPR products can be time-consuming and costly as they need to ensure the efficacy of these products for environmental safety, non-toxic to humans and animals and deliver the claimed benefits (Table 3). Raising awareness for the farming community, providing training, and offering technical support are essential for successfully adopting these products. To be widely adopted, these products must demonstrate clear economic benefits that outweigh their costs. While PGPR can be environmentally friendly compared to certain chemical fertilizers and pesticides, it is crucial to assess the long-term effects of PGPR application on soil health and microbial communities. Scaling up the production of PGPR products to meet the demand of commercial agriculture can be challenging. Establishing an efficient distribution network to reach farmers globally is crucial for successful commercialization. Therefore, addressing these challenges will require continued research, collaboration between researchers and industry, and efforts to create awareness and understanding among farmers and stakeholders.

**Table 3:** Multi-omics approaches associated in combating abiotic stress in plants upon application of PGPR.

PGPR strain	Multi-omics approach	Advancements and findings	References
*Pseudomonas* spp.	Genomics	Identification of stress-responsive genes in *P. fluorescens* for drought and salt tolerance	Cho et al. (2015); Saakre et al. (2017)
	Transcriptomics	Characterization of plant gene expression changes in response to the PGPR under drought and salt stress	Mellidou et al. (2021); Nishu et al. (2022)
	Metabolomics	Profiling of metabolites involved in salt stress mitigation by the PGPR	Mellidou et al. (2021)
	Functional validation	CRISPR-Cas9 knockout of candidate genes to validate the role of PGPR in salt stress tolerance	Chauhan et al. (2022)
*Bacillus* spp.	Proteomics	Identification of salt stress-related proteins produced by *Bacillus* sp.	Zhao et al. (2022)
	Transcriptomics	Examination of plant gene expression changes in response to the PGPR under salt stress	Akbar et al. (2022)
	Metagenomics	Analysis of the rhizosphere microbiome and its interaction with *B. subtilis* for drought tolerance	No et al. (2022)
	Metabolomics	Elucidation of metabolic pathways influenced by *Bacillus* sp. in salt stress condition	Zhao et al. (2022)
*Azospirillum* spp.	Epigenomics	Study of DNA methylation patterns in the presence of *A. brasilense* in response to abiotic stresses	Lephatsi et al. (2021)
	Metagenomics	Studying metagenomics to explain plant growth promoting mechanisms of *A. lipoferum* in drought stress	No et al. (2022)
	Comparative genomics	Comparison of *A. brasilense* genomes to identify stress-related genes	Wiggins et al. (2022)
	Integrative analysis	Systems biology modeling of *A. brasilense*-plant interactions under salt stress	Zuluaga et al. (2022)
*Enterobacter* spp.	Proteomics	Investigation of differential expressed proteins in inducing plant tolerance to salt stress upon *E. cloacae* inoculation	Singh et al. (2017)
	Comparative genomics	Studying the alleviation of salt stress by *Enterobacter* sp.	Kim et al. (2014)
*Rhizobium* spp.	Transcriptomics	Differential drought stress-related gene expression in plants due to inoculation of *R. leguminosarum*	Jiménez-Guerrero et al. (2018); Barquero et al. (2022)
	Functional validation	RNAi-mediated silencing of specific genes to assess the role of *R. leguminosarum* in salt stress resistance	Dong et al. (2013)
*Arthrobacter* spp.	Metabolomics	Identification of metabolites produced by *Arthrobacter* under salt stress condition	Khan et al. (2021a,b)
	Metagenomics	Studying metagenomics to explain plant growth promoting mechanisms of *A. chlorophenolicus* in drought stress	No et al. (2022)
	Comparative genomics	Comparative analysis of *Arthrobacter* sp. genomes to find stress-related genes under drought stress	Chhetri et al. (2022)
	Transcriptomics	Studying to understand how *Arthrobacter* sp. adapts its metabolism in response to PEG-induced drought stress	Gabriel et al. (2022)

Enhancing the application of PGPR under stressful conditions is a promising avenue for sustainable agriculture, and tailoring PGPR formulations and applications to specific stress types requires a deeper understanding of stress-specific mechanisms. Transcriptomics, proteomics, and metabolomics can be employed to analyze the plant-PGPR interaction under particular stress conditions. Integrating these omics data can provide insights into the stress-responsive genes, proteins, and metabolites involved, thereby facilitating the development of stress-tailored PGPR products. Not all PGPR strains exhibit the same level of stress tolerance, limiting their effectiveness under harsh conditions, and the application of genomics and metagenomics can help identify stress-tolerant PGPR strains from diverse microbial communities in the rhizosphere. In addition, metatranscriptomics and metaproteomics can assess changes in the gene expression and protein profiles of PGPR under stress. The above knowledge can guide the development of stress-adaptive PGPR formulations that maintain activity and viability during adverse conditions. The signaling pathways between PGPR and plants are complex and can be altered under stress, affecting communication and beneficial outcomes.

Similarly, phosphoproteomics and epigenomics can help unravel the changes in signaling pathways between PGPR and plants under stress. Understanding these modifications can enable the fine-tuning of PGPR formulations to enhance crop stress tolerance and growth promotion. Further, stress conditions will alter the soil microbial community, potentially influencing the interaction between PGPR and other microorganisms, and these changes can be assessed through metagenomics and 16S rRNA sequencing. Translating lab-scale findings to field conditions can be challenging, and monitoring PGPR performance in large-scale agricultural practice is essential for their practical applications. Besides, remote sensing technologies, combined with transcriptomics and metabolomics analyses of plant samples from various field sites, can enable real-time monitoring of PGPR-mediated responses under diverse stressful conditions. This integrated approach can help optimize PGPR application strategies for different crops and environments. By addressing these key challenges through multi-omics approaches, we can enhance the commercial application of PGPR under stressful conditions and unlock their full potential in sustainable agriculture.

## 7. Conclusion

In an era marked by climate unpredictability and the ever-increasing demand for agricultural productivity, harnessing the power of beneficial microbes like PGPR is emerging as a pivotal approach to enhance plant stress resilience. The review highlights the salient strategies and recent advancements in manipulating PGPR to combat the detrimental effects of stress on crop plants. Recent research underscores the role of PGPR in modulating root architecture that enable plants to explore a larger soil volume, deeper resources and establish a more efficient nutrient and water absorption system. The synergy between PGPR and plants is increasingly recognized as a powerful mechanism for stress mitigation wherein the partnership enhances nutrient uptake and bolsters the plant’s ability to withstand adverse conditions. Emerging technologies like the multi-omics approach and synthetic biology hold promise for tailoring PGPR strains and optimizing their performance in addition to integrating PGPR into precision agriculture systems, leading to more targeted and efficient stress management. In conclusion, the symbiotic relationship between PGPR and plants offers an exciting avenue for agricultural sustainability in the face of mounting environmental challenges. By strategically selecting and applying PGPR strains alongside complementary stress management practices, we can empower crops to thrive in stressed environments. These strategies and innovations might be explored in plant-microbe interactions as we move closer to a future where resilient crops stand as a safeguard against the uncertainties of climate change and global food security.

## Author contributions

MM and RS: conceptualization, supervision, and writing—original draft. AO, MR, and AA-T: data curation, formal analysis, writing—review and editing, and fund acquisition. All authors contributed to the article and approved the submitted version.

## Funding

This research was funded by the Deputyship for Research and innovation, Ministry of Education, Saudi Arabia for funding this research work through the project number (Qu-IF-1-1-1).

## Conflict of interest

The authors declare that the research was conducted in the absence of any commercial or financial relationships that could be construed as a potential conflict of interest.

## Publisher’s note

All claims expressed in this article are solely those of the authors and do not necessarily represent those of their affiliated organizations, or those of the publisher, the editors and the reviewers. Any product that may be evaluated in this article, or claim that may be made by its manufacturer, is not guaranteed or endorsed by the publisher.
